# *QuickStats:* Percentage* of Emergency Department (ED) Visits Made by Adults with Influenza and Pneumonia^†^ That Resulted in Hospital Admission, by Age Group — United States, 2017–2018^§^

**DOI:** 10.15585/mmwr.mm6949a6

**Published:** 2020-12-11

**Figure:**
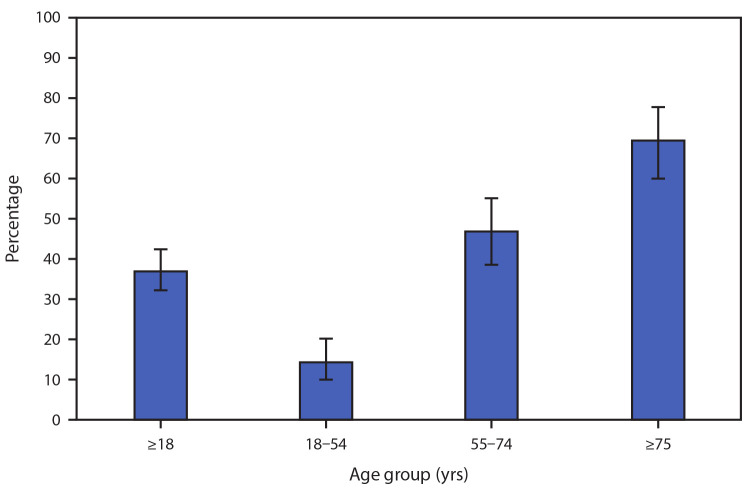
During 2017–2018, 37.2% of ED visits for influenza and pneumonia by adults aged ≥18 years resulted in a hospital admission. The percentage increased with age from 14.4% for adults aged 18–54 years to 46.9% for adults aged 55–74 years and 69.7% for adults aged ≥75 years.

**Source:** National Center for Health Statistics. National Hospital Ambulatory Medical Care Survey, 2017–2018. https://www.cdc.gov/nchs/ahcd/ ahcd_questionnaires.htm.

